# Type 2 Diabetes Is Associated With Increased Complications and Mortality After Hip Fracture in Older Adults Aged 60 Years or Older

**DOI:** 10.1111/dom.71000

**Published:** 2026-06-16

**Authors:** Xincheng Zou, Manju Chandran, Xi Xiong, Chi‐Ho Lee, Wing‐Sun Chow, Carol H. Y. Fong, Lawrence C. M. Lau, Ka‐Shing Cheung, Carlos K. H. Wong, Kathryn C. B. Tan, Yu‐Cho Woo, Karen S. L. Lam, David T. W. Lui

**Affiliations:** ^1^ Department of Medicine, School of Clinical Medicine, Li Ka Shing Faculty of Medicine The University of Hong Kong Hong Kong SAR China; ^2^ Osteoporosis and Bone Metabolism Unit, Department of Endocrinology Singapore General Hospital Singapore; ^3^ DUKE NUS Medical School Singapore; ^4^ Asia Pacific Consortium on Osteoporosis (APCO) Hong Kong SAR China; ^5^ Department of Orthopaedics and Traumatology, School of Clinical Medicine, Li Ka Shing Faculty of Medicine The University of Hong Kong Hong Kong SAR China; ^6^ School of Public Health, Li Ka Shing Faculty of Medicine The University of Hong Kong Hong Kong SAR China; ^7^ Diabetes Research Centre University of Leicester Leicester UK

**Keywords:** diabetes mellitus, epidemiology, fractures, osteoporosis

## Abstract

**Aims:**

Type 2 diabetes (T2D) is associated with increased hip fracture risk. We evaluated the impact of T2D on post‐hip fracture outcomes, which is less well described.

**Materials and Methods:**

Individuals aged ≥ 60 years hospitalised for hip fractures between 2011 and 2020 were identified from the territory‐wide electronic health records of Hong Kong and stratified by their diabetes status at the time of hip fractures. Individuals were followed up from day of admission to outcomes, death, or December 2023, whichever was earlier. Outcomes included in‐hospital complications, in‐hospital and 1‐year mortality, length of hospitalisation, costs, anti‐osteoporosis treatment rate and recurrent fractures. Multivariable regression analyses were performed to identify the independent associations between diabetes and various outcomes. We further analysed the subgroup with undiagnosed diabetes for its impact on post‐hip fracture outcomes.

**Results:**

We identified 52 472 individuals who sustained hip fractures (34.6% had pre‐existing diabetes). T2D was independently associated with more cardiovascular, kidney and infective complications during hospitalisation (all *p* < 0.001), and higher 1‐year mortality (adjusted HR 1.08, 95% CI 1.04–1.13). While in‐hospital mortality and length of stay were not significantly different, hospitalisation costs were 4.6% higher in T2D (*p* < 0.001). Individuals with undiagnosed diabetes showed even higher risks of in‐hospital complications and mortality, along with 24.5% higher costs (*p* < 0.001). T2D did not influence the rate of anti‐osteoporosis treatment or recurrent fracture risks.

**Conclusions:**

T2D was associated with worse post‐hip fracture outcomes and increased healthcare burdens. Early T2D diagnosis and management, coupled with early attention to bone fragility in T2D, is warranted.

## 1 Introduction

Both diabetes and osteoporosis are prevalent globally [1, 2]. The dreaded complications of osteoporosis are fragility fractures. Among these, hip fractures are one of the most serious types, bringing about significant morbidity and mortality [3]. Studies, including work from our group, have consistently demonstrated the higher fracture risk in type 2 diabetes [4, 5]. Earlier studies have suggested increased complications [6, 7] and mortality [8] among hip fracture patients comorbid with type 2 diabetes. With the expanded choices of pharmacotherapy in type 2 diabetes resulting in better glycaemic control [9], it is relevant to examine using a large territory‐wide electronic health database whether the excess post‐fracture mortality related to diabetes improved over time. Data are also limited regarding the excess of hospitalisation costs associated with diabetes, which will inform public health policy for prioritising management of bone fragility in type 2 diabetes. Moreover, existing literature focuses on pre‐existing diabetes, so how undiagnosed diabetes impacts all the above post‐fracture outcomes remains to be elucidated.

Optimising treatment rate of osteoporosis is another key aspect to address bone fragility in type 2 diabetes. Studies in Denmark and the United States have identified a lower likelihood of anti‐osteoporosis treatment following major osteoporotic fractures (MOF), but not when stratified by specific sites [10, 11]. This could be because type 2 diabetes is associated with higher bone mineral density (BMD) than non‐diabetes counterparts, such that adopting the usual BMD T‐score threshold for patients with type 2 diabetes may lead to undertreatment. Nonetheless, hip fracture per se is an indication for anti‐osteoporosis treatment regardless of BMD according to international guidelines [12]. Examining the differences in anti‐osteoporosis treatment rate and subsequent fracture risk by diabetes status specifically among patients who sustained fragility hip fractures helps identify care gaps and formulate focused quality improvement strategies.

Hence, we conducted a retrospective territory‐wide cohort study in Hong Kong to comprehensively examine the impact of type 2 diabetes on clinical outcomes, medical costs, and osteoporosis treatment following hip fractures.

## 2 Materials and Methods

### 2.1 Data Source

We conducted a retrospective study using the territory‐wide pseudonymised electronic health records from the Hong Kong Hospital Authority (HA). HA is a statutory body that provides public medical services and manages all public hospitals in Hong Kong. The service is available to all Hong Kong residents (a population of > 7.5 million), and covers 90% of all secondary and tertiary care in the territory [13]. Most patients with chronic diseases are managed under HA because of high government subsidies [14]. The database in HA is a comprehensive capture of the public hospital system's data (including diagnoses, investigations and treatments) for all individuals who have ever attended outpatient and inpatient services in the public hospital system [15]. Fasting glucose (FG) and HbA1c are readily available in Hong Kong for screening of diabetes. Furthermore, comprehensive management programmes are available in HA for patients diagnosed with diabetes [13], including regular diabetic complication screening [16]. During each diabetic complication screening session, patients are assessed clinically and have laboratory investigations to determine their control of diabetes, its related cardiovascular risk factors and the presence of diabetic complications. This database has been commonly used to conduct population‐based studies related to diabetes [5, 14, 17, 18] and fragility fractures [5, 14, 18] with validated diagnostic codes for identifying fracture outcomes.

### 2.2 Study Population

We identified all individuals aged ≥ 60 years hospitalised for hip fractures in Hong Kong between 1 January 2011 and 31 December 2020. Exclusion criteria were: (i) prior history of hip fractures, and (ii) patients who sustained hip fractures from high‐energy trauma during the same episode (identified by ICD‐9‐CM codes E800‐7, E810‐31, and E840‐8). Among individuals who had more than one episode of hip fracture during the inclusion period, only the first episode was included.

The index date was the date of admission for hip fractures. Patients were followed up until the occurrence of outcomes, death, or 31 December 2023, whichever was earlier.

Individuals were classified as having diabetes if they met one or more of the following before the index date: presence of ICD‐9‐CM code 250, FG ≥ 7.0 mmol/L, HbA1c ≥ 6.5% (48 mmol/mol), or prescription of anti‐diabetic medications. The earliest date satisfying the diagnostic criteria was considered the date of diagnosis of diabetes. As this study focuses on the impact of type 2 diabetes on post‐hip fracture outcomes, patients diagnosed to have type 1 diabetes were excluded based on ICD‐9‐CM codes and physician‐diagnosed type 1 diabetes (incorporating clinical history, biochemical and/or immunological findings).

### 2.3 Study Outcomes

The following complications were identified during the hospitalisation for each individual: (i) infections, including septicaemia (defined by ICD‐9‐CM 038 and 998.5), pneumonia (defined by ICD‐9‐CM 480–487), urinary tract infection (ICD‐9‐CM 599.0), and deep wound infection (ICD‐9‐CM 584.9); (ii) acute renal failure (ICD‐9‐CM 584.9); (iii) acute ischaemic stroke (ICD‐9‐CM 433–438); (iv) acute myocardial infarction (ICD‐9‐CM 410); and (v) acute pulmonary embolism (ICD‐9‐CM 415.1).

We evaluated the initiation of anti‐osteoporosis treatment and recurrent fractures at 2 years after the index hip fracture in view of the relevance of imminent fracture risk [19]. To capture the rate of anti‐osteoporosis treatment following hip fracture, prescriptions for anti‐osteoporosis medications were retrieved, including bisphosphonates (excluding intravenous bisphosphonates for primary or secondary bone tumours), denosumab (Prolia; excluding Xgeva), raloxifene, strontium, teriparatide and romosozumab. Abaloparatide is not available in Hong Kong. Recurrent fractures following discharge from the index hip fracture hospitalisation were identified, including fractures of the spine and trunk (ICD‐9‐CM codes 805–809), upper limb (ICD‐9‐CM codes 810–814, 817–819), and lower limb (ICD‐9‐CM codes 820–824, 827–829). Any fracture cases attributed to accidental injuries (ICD‐9‐CM codes E800‐E807, E810‐E831, E840‐E848) were not counted as recurrences. To minimise potential overestimation of recurrent fracture events, each fracture event required a 365‐day washout period from the previous episode of fracture at the same site. Specifically, recurrent hip fractures were defined as subsequent hospitalisation for hip fracture (ICD‐9‐CM code 820) accompanied by a related surgical procedure (ICD‐9‐CM procedure codes 78.55, 79.15, 79.35, 81.51–53) in that episode.

Prolonged hospitalisation was defined as hospital stay ≥ 30 days. Costs of hospitalisations were calculated by the sum of those of operations, procedures and hospital stay recorded on the discharge summary. The costs of items were taken with reference to information available on the HA webpage [20]. To facilitate interpretation of the healthcare burden, we broke down the costs into those for (i) hospitalisation in general wards, (ii) procedures, and (iii) intensive care.

### 2.4 Definition of Covariates

The following covariates were retrieved: obesity (defined by ICD‐9‐CM code 278) and history of MOF (clinical vertebral and upper limb fractures; ICD‐9‐CM 805, 812–814). The definitions of components of Charlson comorbidity index (CCI) are listed in Table S1. As our analysis focuses on the impact of diabetes on various outcomes, CCI was modified (termed ‘modified CCI’ thereafter) to exclude the presence and severity of diabetes in the scoring as this was treated separately as an independent variable in this study [6].

### 2.5 Statistical Analyses

All analyses were performed using R (Version 4.4.1). Two‐sided *p*‐value < 0.05 was considered statistically significant. For categorical variables, descriptive statistics were reported as counts with percentages. For continuous variables, normality was assessed using the Shapiro–Wilk test. Variables were reported as medians with interquartile ranges (IQR) as they did not follow normal distribution. Between‐group comparisons were performed using the Wilcoxon rank‐sum test for continuous variables and the Chi‐square test for categorical variables.

We tested the temporal trend of various post‐fracture outcomes over the decade of 2011–2020 by logistic or linear regression analyses as appropriate to test for the p‐for‐trend. The dependent variable was the respective post‐fracture outcomes, while the independent variable was the year of admission.

Multivariable logistic regression analysis was performed to examine whether diabetes was independently associated with in‐hospital complications. Multivariable Cox regression was used to assess the independent association of diabetes with in‐hospital and 1‐year mortality. For outcomes where non‐fatal events were of interest, Fine‐Grey competing risk models were employed considering death as a competing event to evaluate the association between diabetes and prolonged hospitalisation, 2‐year anti‐osteoporosis treatment initiation, and 2‐year fracture recurrence. Sex, age, obesity, modified CCI, and admission period (2011–2015 vs. 2016–2020) were adjusted in all models. In the analysis of fracture recurrence, we additionally adjusted for baseline use of anti‐osteoporosis medication and prevalent MOF. For the analysis of factors associated with anti‐osteoporosis treatment initiation, we excluded patients with baseline prescription of anti‐osteoporosis medication and additionally adjusted for prevalent MOF. To determine whether medication adherence contributed to the association between anti‐osteoporosis treatment and refracture risk, medication possession ratio (MPR) was calculated as a proxy for adherence among patients who were initiated on anti‐osteoporosis agents within 2 years after hip fracture. We then compared MPR (as a continuous measure) between patients with diabetes and those without diabetes using the Wilcoxon rank‐sum test.

Multivariable linear regression analysis was used to assess the independent association between diabetes and hospitalisation cost, adjusted for sex, age, obesity, modified CCI and year of admission. The hospitalisation cost was logarithmically transformed before analysis. To estimate the excess of hospitalisation cost associated with diabetes, the beta‐coefficient associated with diabetes in the multivariable model was transformed using the equation = $\left(e^{\beta }-1\right)\times 100\%$.

### 2.6 Subgroup Analyses

First, we stratified patients with pre‐existing diabetes into three groups based on the number of classes of anti‐diabetic medications prescribed (0, 1, or ≥ 2) and examined the association between each group and various post‐fracture outcomes with reference to the group without diabetes. Second, among individuals not fulfilling the definition of diabetes before the index date, we identified a subgroup who had undiagnosed diabetes (i.e., those whose date of diabetes diagnosis occurred between their hip fracture admission and discharge dates) to examine the association between undiagnosed diabetes and various post‐fracture outcomes, with reference to the pre‐existing diabetes and non‐diabetes subgroups. Furthermore, the subgroup of undiagnosed diabetes was further stratified into (i) pre‐existing unrecognised diabetes (defined by presence of ICD‐9‐CM code 250, HbA1c ≥ 6.5%, or prescription of anti‐diabetic medications between hip fracture admission and discharge date); (ii) fracture‐induced stress hyperglycaemia (defined by fasting glucose ≥ 7.0 mmol/L without fulfilling criteria for pre‐existing unrecognised diabetes in the 2 years post‐fracture); and (iii) newly detected metabolic disorders (defined by fasting glucose ≥ 7.0 mmol/L without fulfilling criteria for pre‐existing unrecognised diabetes during hospitalisation but subsequently developed diabetes in the 2 years post‐fracture) to examine the association between these hyperglycaemic entities and various post‐fracture outcomes with reference to the pre‐existing diabetes and non‐diabetes subgroups.

### 2.7 Sensitivity Analyses

Several sensitivity analyses were performed to demonstrate the robustness of results. First, we repeated the analyses according to the type of fracture using ICD‐9‐CM codes 820.0–820.1 (transcervical) and 820.2–820.3 (pertrochanteric). Second, we excluded patients with a history of cancer (see Table S1 for the ICD‐9‐CM codes for ‘any malignancy’).

## 3 Results

Figure S1 shows the study flow diagram. In total, 52 472 individuals with hip fractures were included (median age 84 [IQR: 78–89], 67.3% females, and 34.6% having pre‐existing diabetes). Their baseline characteristics are summarised in Table 1. The post‐hip fracture outcomes were summarised in Table 2.

**TABLE 1:** Baseline characteristics of the cohort.

	All	Diabetes	Non‐diabetes
Number of patients	52 472	18 130	34 342
Age (years)	84 (78–89)	83 (77–88)	85 (79–90)
60–69	4273 (8.1)	1498 (8.3)	2775 (8.1)
70–79	11 003 (21.0)	4502 (24.8)	6501 (18.9)
≥ 80	37 196 (70.9)	12 130 (66.9)	25 066 (73.0)
Sex			
Male	17 162 (32.7)	5452 (30.1)	11 710 (34.1)
Female	35 310 (67.3)	12 678 (69.9)	22 632 (65.9)
Obesity	500 (1.0)	345 (1.9)	155 (0.5)
Admission year			
2011–2015	26 093 (49.7)	8326 (45.9)	17 767 (51.7)
2016–2020	26 379 (50.3)	9804 (54.1)	16 575 (48.3)
Modified Charlson comorbidity index	1 (0–2)	1 (0–2)	1 (0–2)
0	21 419 (40.8)	6501 (35.9)	14 918 (43.4)
1	13 036 (24.8)	4267 (23.5)	8769 (25.5)
2	8797 (16.8)	3307 (18.2)	5490 (16.0)
≥ 3	9220 (17.6)	4055 (22.4)	5165 (15.0)
History of anti‐osteoporosis treatment	2375 (4.5)	728 (4.0)	1647 (4.8)
History of major osteoporotic fractures	6333 (12.1)	2237 (12.3)	4096 (11.9)

*Note:* Data presented as median (interquartile range) or number (percentage).

**TABLE 2:** Post‐fracture outcomes of the entire cohort of patients with hip fracture.

Outcome	All	Diabetes	Non‐diabetes
Length of hospitalisation (days)	27 (18–37)	28 (19–38)	27 (18–37)
≤ 30 days	31 028 (59.1%)	10 255 (56.6%)	20 773 (60.5%)
> 30 days	21 444 (40.9%)	7875 (43.4%)	13 569 (39.5%)
Mortality			
During hospitalisation	1605 (3.1%)	546 (3.0%)	1059 (3.1%)
Within 1 year	8881 (16.9%)	3238 (17.9%)	5643 (16.4%)
Anti‐osteoporosis initiation in 2 years	9681 (19.3%)	3415 (19.6%)	6266 (19.2%)
Recurrent fractures in 2 years	3446 (6.6%)	1202 (6.6%)	2244 (6.5%)
Cost (HKD)	227 210 (172942–290 495)	232 200 (177350–297 341)	226 235 (171260–287 600)

*Note:* Results presented as median (IQR) or number (percentage).

### 3.1 Trend of Post‐Fracture Outcomes in 2011–2020

Table 3 shows the secular trend of post‐fracture outcomes in 2011–2020. Over the decade, the proportion of individuals who developed post‐hip fracture complications during hospitalisation decreased (*p* < 0.001), while the in‐hospital mortality (*p* = 0.002) and the median length of hospitalisation increased (*p* < 0.001). Nonetheless, 1‐year mortality (*p* = 0.21) and recurrent fractures at 2 years after hip fractures (*p* = 0.43) remained static.

**TABLE 3:** Secular trends of post‐hip fracture outcomes.

Year	Occurrence of in‐hospital complications (%)	In‐hospital mortality (%)	1‐year mortality (%)	Length of hospitalisation (days)	Anti‐osteoporosis initiation at 2 years (%)	Recurrent fractures at 2 years (%)
2011	22.2	2.9	17.2	27	13.7	4.0
2012	21.2	2.9	16.8	27	12.3	4.0
2013	17.5	2.6	16.9	27	13.7	3.7
2014	15.6	3.2	18.1	26	15.1	4.0
2015	16.1	2.6	16.5	27	15.8	3.6
2016	15.4	3.1	16.4	28	16	3.9
2017	13.4	3.0	16.5	28	19.8	4.1
2018	12.8	3.2	16.5	28	23.9	4.0
2019	10.3	3.3	16.6	29	30.5	3.8
2020	12.8	3.9	17.8	27	34.1	3.6
*p* for trend	< 0.001	0.002	0.21	< 0.001	< 0.001	0.43

### 3.2 Impact of Diabetes on Post‐Hip Fracture Morbidity and Mortality

Patients with diabetes had higher rates of in‐hospital complications than those without diabetes: namely, infective, cardiovascular and kidney complications (all *p* < 0.001, Table 4). Diabetes was independently associated with higher risks of in‐hospital complications (adjusted OR [aOR]: 1.16, 95% CI: 1.10–1.22, *p* < 0.001), septicaemia (aOR: 1.23, 95% CI: 1.07–1.41, *p* = 0.003), acute renal failure (aOR: 1.97, 95% CI: 1.60–2.41, *p* < 0.001), acute ischaemic stroke (aOR: 1.21, 95% CI: 1.11–1.31, *p* < 0.001), and acute myocardial infarction (aOR: 1.33, 95% CI: 1.14–1.55, *p* < 0.001) (Table 4). Events of acute pulmonary embolism were too few to reveal any significant difference between diabetes and non‐diabetes groups (Table 4). Diabetes was independently associated with 1‐year mortality (aHR 1.06, 95% CI 1.02–1.11, *p* = 0.01), but not an independent factor of in‐hospital mortality (aHR 0.98, 95% CI 0.95–1.03, *p* = 0.50) or prolonged hospitalisation (aHR: 0.99, 95% CI: 0.96–1.01, *p* = 0.31) (Table 5).

**TABLE 4:** Risks of various complications during hospitalisation for hip fractures among patients with and without diabetes.

	Number of patients	Events	Incidence	Adjusted OR^a^	95% CI	*p*
Any in‐hospital complications						
No diabetes	34 342	5146	15.0%	Reference		
Diabetes	18 130	3098	17.1%	1.16	1.10–1.22	< 0.001
Infective complications						
No diabetes	34 342	3245	9.4%	Reference		
Diabetes	18 130	1830	10.1%	1.10	1.03–1.17	0.002
Septicaemia						
No diabetes	34 342	563	1.6%	Reference		
Diabetes	18 130	364	2.0%	1.23	1.07–1.41	0.003
Acute renal failure						
No diabetes	34 342	185	0.5%	Reference		
Diabetes	18 130	193	1.1%	1.97	1.60–2.41	< 0.001
Acute ischaemic stroke						
No diabetes	34 342	1725	5.0%	Reference		
Diabetes	18 130	1168	6.4%	1.21	1.11–1.31	< 0.001
Acute myocardial infarction						
No diabetes	34 342	416	1.2%	Reference		
Diabetes	18 130	294	1.6%	1.33	1.14–1.55	< 0.001
Acute pulmonary embolism						
No diabetes	34 342	134	0.4%	Reference		
Diabetes	18 130	64	0.4%	0.92	0.68–1.25	0.59

Abbreviations: CI, confidence interval; OR, odds ratio.

^a^ Adjusted for sex, age, obesity, modified Charlson comorbidity index, and year of admission.

**TABLE 5:** Independent association between diabetes and various post‐hip fractures outcomes.

	Crude HR (95% CI)	*p*	Adjusted HR (95% CI)	*p*
Prolonged hospitalisation	0.99 (0.97–1.02)	0.46	0.99 (0.96–1.01)	0.31^a^
Mortality during hospitalisation	0.91 (0.82–1.01)	0.08	0.93 (0.83–1.03)	0.14^a^
Mortality within 1 year	1.09 (1.05–1.14)	< 0.001	1.06 (1.02–1.11)	0.01^a^
Initiation of anti‐osteoporosis treatment in 2 years (*n* = 50 097)	1.03 (0.99–1.07)	0.22	0.98 (0.95–1.03)	0.50^b^
Recurrent fractures in 2 years	1.02 (0.95–1.09)	0.66	1.00 (0.93–1.08)	0.96^c^

Abbreviations: CI, confidence interval; HR, hazards ratio.

^a^ Adjusted for sex, age, obesity, modified Charlson comorbidity index, and year of admission.

^b^ Adjusted for sex, age, obesity, modified Charlson comorbidity index, year of admission, and history of major osteoporotic fractures.

^c^ Adjusted for sex, age, obesity, modified Charlson comorbidity index, year of admission, history of osteoporosis treatment and history of major osteoporotic fractures.

### 3.3 Hospitalisation Costs: Trend in 2011–2020 and Impact of Diabetes

Over the decade, hospitalisation costs per hip fracture episode significantly increased from HKD 224800 (IQR: 169400–287 600) in 2011–2015 to HKD 231080 (IQR: 176800–293 140) in 2016–2020 (*p* < 0.001). The median hospitalisation cost per episode was higher in the diabetes group (HKD 232200, IQR: 177350–297 341) than the non‐diabetes group (HKD 226235; IQR: 171260–287 600) (*p* < 0.001). In the multivariable linear regression analysis, diabetes was an independent factor associated with hospitalisation (beta coefficient = 0.05, *p* < 0.001), representing an excess cost of 4.7% attributable to diabetes. This trend was primarily driven by increased costs for hospitalisation in general wards (+6.53%, *p* < 0.001) and intensive care (+5.29%, *p* = 0.003), while costs for procedures were comparable. Notably, there is a 24.5% excess in total costs for patients with undiagnosed diabetes (*p* < 0.001): driven by a 31.6% increase in costs for hospitalisation in general wards (*p* < 0.001) and a 38.1% increase in costs for intensive care (*p* = 0.01) while costs for procedures were comparable.

### 3.4 Post‐Hip Fracture Osteoporosis Management in 2011–2020 and Impact of Diabetes

Among 50 097 patients with no prior anti‐osteoporosis medication, 9681 (19.3%) initiated treatment within 2 years of index hip fracture (Table 2). Treatment initiation rates increased significantly over the study period (*p* < 0.001) (Table 3).

In total, 3446 patients (6.6%) experienced recurrent fractures within 2 years (Table 2). Over the decade, the rate of recurrent fractures remained relatively static (*p* = 0.43) (Table 3). There was no significant difference in the rates of recurrent fractures between the diabetes and non‐diabetes groups: in the non‐diabetes group, 2244 patients (6.5%) had recurrent fractures within 2 years; and 1202 patients (6.6%) in the diabetes group.

In the multivariable Cox regression analyses, diabetes was not an independent factor of anti‐osteoporosis medication initiation after index fracture (*p* = 0.50) nor recurrent fractures (*p* = 0.96) (Table 5). The median MPR of anti‐osteoporosis medications was comparable between the diabetes (68.0%, IQR 38.3–91.1) and non‐diabetes groups (65.1%, IQR 36.7–90.3, *p* = 0.12).

### 3.5 Subgroup Analyses

Upon stratifying patients with pre‐existing diabetes into three groups according to the number of classes of anti‐diabetic medications, results were consistent with the main analyses. Moreover, there was a trend of higher odds of post‐fracture adverse outcomes with increasing number of classes of anti‐diabetic medication use—an indirect reflection of the complexity of diabetes (Tables S2 and S3).

Among patients without pre‐existing diabetes, 0.6% (*n* = 210) were identified with undiagnosed diabetes. Upon identifying this separate group of patients with undiagnosed diabetes, findings comparing patients with pre‐existing diabetes (*n* = 18 130) to those without diabetes (*n* = 34 342) remained consistent (Tables S4 and S5). Compared to patients without diabetes, those with undiagnosed diabetes as a whole had significantly higher risks of in‐hospital complications (aOR 2.49, 95% CI 1.79–3.47, *p* < 0.001), in‐hospital mortality (aHR 1.86, 95% CI 1.10–3.17, *p* = 0.02), and 1‐year mortality (aHR 1.46, 95% CI 1.07–2.01, *p* = 0.02), despite being less likely to have a prolonged hospital stay (aHR 0.74, 95% CI 0.63–0.86, *p* < 0.001). Direct comparison with patients who had pre‐existing diabetes revealed that the undiagnosed group still had a significantly elevated risk for in‐hospital complications (aOR 2.15, 95% CI 1.54–3.00, *p* < 0.001) and in‐hospital mortality (aHR 1.94, 95% CI 1.14–3.33, *p* = 0.02), in addition to a similarly shorter hospital stay (aHR 0.75, 95% CI 0.64–0.87, *p* < 0.001) (Tables S4 and S5). However, no significant differences were observed between the three groups in terms of anti‐osteoporotic treatment initiation or recurrent fractures within 2 years.

This group of 210 patients with undiagnosed diabetes mentioned above was further stratified into patients with pre‐existing unrecognised diabetes (*n* = 82), fracture‐induced stress hyperglycaemia (*n* = 121), and newly detected metabolic disorders (*n* = 7). Due to the limited number of patients classified as newly detected metabolic disorders, we only compared the subgroups of pre‐existing unrecognised diabetes and fracture‐induced stress hyperglycaemia with non‐diabetes and pre‐existing diabetes, where results were generally consistent with the main analyses (Tables S6 and S7).

### 3.6 Sensitivity Analyses

In the first sensitivity analysis stratified by fracture type, the associations between diabetes and adverse outcomes were consistently observed in the subgroup with transcervical femoral fracture (Tables S8 and S9) and subgroup with pertrochanteric femoral fracture (Tables S10 and S11). In the second sensitivity analysis after excluding 4637 (8.8%) patients with a history of cancer, results remained consistent (Tables S12 and S13).

## 4 Discussion

Our territory‐wide study provided an updated landscape of morbidity and mortality contributed by diabetes among 52 472 patients with hip fractures over the second decade of the 21st century, offering insights by additionally evaluating the associated healthcare costs and anti‐osteoporosis treatment rates. Over the decade, the rate of post‐hip fracture complications and mortality during hospitalisation has improved, although 1‐year mortality after hip fractures has yet to show improvements. Pre‐existing diabetes was independently associated with increased risk of complications and 1‐year mortality, leading to increased costs of hospitalisation. Notably, undiagnosed diabetes was associated with even higher risks of in‐hospital complications, mortality and hospitalisation costs. Although in‐hospital mortality was similar between patients with pre‐existing diabetes and non‐diabetic patients, a notably higher risk was observed in those with undiagnosed diabetes. We observed an encouraging rise in the anti‐osteoporosis medication initiation post‐fracture, though only up to around 34% at most. Yet, the recurrent fracture rates remained static. Furthermore, diabetes status showed no independent association with either treatment initiation or recurrent fracture risk.

Despite previous studies showing the overall improving glycaemic control in the territory in recent years [9], diabetes was still independently associated with adverse outcomes during hospitalisation and 1‐year mortality. In fact, cardiovascular and kidney complications are two key contributors to post‐hip fracture complications [21], echoed by the higher adjusted odds of the diabetes group for cardiovascular and kidney complications in our study. This suggests the need for comprehensive care for cardiovascular‐kidney‐metabolic risks for patients with type 2 diabetes.

While all existing studies focused on the association between pre‐existing diabetes and various post‐hip fracture outcomes [6, 7, 22], we found that patients with undiagnosed diabetes had an even higher risk of developing complications during hospitalisation than those with pre‐existing diabetes. This is in line with a study in a general cohort of hospitalised patients showing that inpatient hyperglycaemia, especially newly diagnosed hyperglycaemia, was associated with poor clinical outcomes during hospitalisation [23]. Other studies, often in cardiology patients, showed that undiagnosed diabetes was associated with poorer outcomes [23, 24, 25]. Given that undiagnosed diabetes is associated with worse post‐fracture outcomes and that diabetes is a common secondary cause of osteoporosis [26], routine assessment of glycaemic status in patients hospitalised for hip fractures may help identify those at higher risk for adverse events. One should be aware of fracture‐induced stress hyperglycaemia—a phenomenon seen in previous studies [27], which may complicate the interpretation of the glycaemic status of the patients concerned. Its presence means that further surveillance for the glycaemic status after the resolution of stress is necessary before embarking on long‐term intervention. Further work is warranted to evaluate the extent fracture stress worsens glycaemia using a cohort of patients without diabetes and stratified by exposure to fracture and non‐exposure. Nonetheless, routine assessment of glycaemic status can help to identify a high‐risk group of patients for optimisation of glycaemic control for better post‐fracture outcomes since our study found that both pre‐existing unrecognised diabetes and stress hyperglycaemia were associated with worse post‐fracture outcomes.

The encouraging increasing trend of anti‐osteoporosis treatment prescriptions following hip fractures is likely contributed to by the implementation of multidisciplinary care in the management of hip fractures, notably the orthogeriatric multidisciplinary co‐management programme since late 2018 [28]. Nonetheless, there is still room for improvement since the latest rate of osteoporosis treatment after hip fractures is still only 30%. We did not see any discrepancy in the rate of anti‐osteoporosis treatment prescription between those with pre‐existing diabetes and those without diabetes, in contrast to the previous studies in the United States [11] and Denmark [10] showing that patients with diabetes were less likely to be prescribed anti‐osteoporosis treatment upon discharge. The difference could be because our study focused on patients with hip fracture, which is a sufficient indication to start anti‐osteoporosis treatment regardless of BMD T‐score, a source of fracture risk underestimation in diabetes [29]. The discrepancy could also be due to a lack of matching for comorbidities in the United States study [11] and the sole reliance on diagnostic coding in defining diabetes [10, 11]. However, our results should also be interpreted considering some limitations of our database—such as the definition of obesity based on ICD‐9‐CM codes (instead of BMI) and lack of BMD data—which might have nullified potential differences in the anti‐osteoporosis prescription rate between patients with and without diabetes.

The rate of recurrent fractures did not differ between non‐diabetes and diabetes in general, corroborating with two recent retrospective studies in the United States and Denmark reporting no significant association between type 2 diabetes and fracture risk following hip fracture [11, 30]. The initiation of anti‐osteoporosis treatment might have modified the association between type 2 diabetes and subsequent fracture risks. Moreover, medication adherence was not a contributing factor for the disconnect between anti‐osteoporosis treatment and refracture risk.

Our study quantified the magnitude of excess hospitalisation cost for hip fractures to be 4.6% for patients with pre‐existing diabetes, and an additional 24.5% for patients with undiagnosed diabetes. Our results were in line with a recent report in Taiwan revealing an increased cost of hospitalisation for hip fractures especially among patients with insulin‐treated diabetes, although the exact magnitude was not quantified in that study [7]. The increased risks of post‐fracture complications and the consequent longer hospital stay likely explain the higher average cost of hospitalisation in patients with diabetes. By addressing the above treatment gaps, it is hoped that the excess financial burden associated with diabetes on the healthcare system could be minimised.

There were several limitations in this study. First, as the identification of most components of CCI relied on ICD codes, underestimation of comorbidity burden could not be entirely excluded. Second, ICD‐9‐CM codes were exclusively used throughout the study period because ICD‐9‐CM remains the coding system used in the hospitals managed under the Hong Kong Hospital Authority. The system in the Hong Kong Hospital Authority has not transitioned to ICD‐10‐CM. We acknowledged that ICD‐9‐CM code 820 for hip fracture lacks the details of subtypes offered by ICD‐10‐CM, which may affect fracture severity stratification and external validity. Nonetheless, we conducted sensitivity analyses according to types of femoral neck fractures indicated by ICD‐9‐CM codes (transcervical and pertrochanteric) which yielded consistent results. Third, there was limited information on BMI especially in the non‐diabetes group. Hence, ICD‐9‐CM code of obesity was used as a surrogate, which could lead to under‐detection of obesity. Fourth, the conclusion related to undiagnosed diabetes was based on a relatively small size of subgroup of 210 patients. Fifth, BMD data were lacking in this database. Sixth, we did not have detailed clinic notes to review the exact reason for the prolonged hospitalisation. However, our results suggested that the more prolonged hospitalisation in patients with diabetes was likely contributed by the increased risks of post‐operative medical complications. Last but not least, while FG and HbA1c measurements are readily available in Hong Kong for screening of diabetes, they may not have been performed systematically when patients were admitted for hip fractures. There may be misclassification of patients with undiagnosed diabetes to non‐diabetes, though this misclassification would tend to nullify our study results.

## 5 Conclusion

Pre‐existing diabetes was independently associated with an increased risk of complications and 1‐year mortality, leading to increased costs of hospitalisation. Notably, undiagnosed diabetes was associated with even higher risks for in‐hospital and 1‐year mortality, complications, and costs. Addressing the cardiovascular‐kidney‐metabolic risk profile may help to reduce the adverse impact of diabetes on post‐fracture outcomes. Diabetes—whether diagnosed or undiagnosed—was not independently associated with the initiation of anti‐osteoporosis treatment or recurrent fractures. We observed an encouraging rise in the proportion of patients with hip fractures who initiated anti‐osteoporosis medications within 2 years, up to around 34%. However, this increased treatment did not translate to decreasing recurrent fracture rates over the years. More proactive measures are therefore necessary to improve both the uptake and effectiveness of osteoporosis management after fracture.

## Author Contributions

X.Z. and D.T.W.L. wrote the first draft of the manuscript. All authors researched data and contributed to discussion. X.Z., X.X., C.H.Y.F. and D.T.W.L. conducted the analyses and interpreted the results; all authors reviewed the manuscript and gave critical input. D.T.W.L. conceived the research idea, is the guarantor of this work and, as such, had full access to all the data in the study and takes responsibility for the integrity of the data and the accuracy of the data analysis. All authors approved the final version of the manuscript.

## Funding

This study received funding support from the Health and Medical Research Fund, the Health Bureau of the Hong Kong SAR Government (ref no. 20211971). This funding source had no role in the design of this study and did not have any role during its execution, analyses, interpretation of the data, or decision to submit results.

## Conflicts of Interest

The authors declare no conflicts of interest.

## Supporting information


**Figure S1:** Study flow chart.
**Table S1:** ICD‐9‐CM Codes for identification of components in the modified Charlson comorbidity index.
**Table S2:** Risks of in‑hospital complications after hip fracture among patients without and with pre‑existing diabetes, where patients with pre‐existing diabetes were stratified by the number of classes of anti‑diabetic medications used.
**Table S3:** Independent factors associated with post‐hip fractures outcomes among patients without and with pre‑existing diabetes, where patients with pre‐existing diabetes were stratified by the number of classes of anti‑diabetic medications used.
**Table S4:** Risks of various complications during hospitalisation for hip fractures among non‐diabetes, pre‐existing diabetes and undiagnosed diabetes.
**Table S5:** Independent factors associated with post‐hip fractures outcomes among non‐diabetes, pre‐existing diabetes and undiagnosed diabetes.
**Table S6:** Risks of various complications during hospitalisation for hip fractures among non‐diabetes, pre‐existing diabetes, pre‐existing undiagnosed diabetes, and fracture induced stress hyperglycaemia.
**Table S7:** Independent factors associated with post‐hip fractures outcomes among non‐diabetes, pre‐existing diabetes, pre‐existing undiagnosed diabetes, and fracture induced stress hyperglycaemia.
**Table S8:** Risks of various complications during hospitalisation for hip fractures (transcervical type; ICD‐9 820.0–1) among patients with and without diabetes.
**Table S9:** Independent association between diabetes and various post‐hip fractures outcomes among patients with hip fractures (transcervical type; ICD‐9 820.0–1).
**Table S10:** Risks of various complications during hospitalisation for hip fractures (pertrochanteric type; ICD‐9 820.2–3) among patients with and without diabetes.
**Table S11:** Independent association between diabetes and various post‐hip fractures outcomes among patients with hip fractures (pertrochanteric type; ICD‐9 820.2–3).
**Table S12:** Risks of various complications during hospitalisation for hip fractures among patients with and without diabetes (excluding patients with a history of cancer).
**Table S13:** Independent association between diabetes and various post‐hip fractures outcomes (excluding patients with a history of cancer).

## Data Availability

The data that support the findings of this study are available from the corresponding author upon reasonable request.

## References

[1] H. Sun , P. Saeedi , S. Karuranga , et al., “IDF Diabetes Atlas: Global, Regional and Country‐Level Diabetes Prevalence Estimates for 2021 and Projections for 2045,” Diabetes Research and Clinical Practice 183 (2022): 109119, 10.1016/j.diabres.2021.109119. 34879977 PMC11057359

[2] J. E. Compston , M. R. McClung , and W. D. Leslie , “Osteoporosis,” Lancet 393, no. 10169 (2019): 364–376, 10.1016/S0140-6736(18)32112-3. 30696576

[3] N. Veronese and S. Maggi , “Epidemiology and Social Costs of Hip Fracture,” Injury 49, no. 8 (2018): 1458–1460, 10.1016/j.injury.2018.04.015. 29699731

[4] M. Zoulakis , L. Johansson , H. Litsne , K. Axelsson , and M. Lorentzon , “Type 2 Diabetes and Fracture Risk in Older Women,” JAMA Network Open 7, no. 8 (2024): e2425106, 10.1001/jamanetworkopen.2024.25106. 39106069 PMC11304123

[5] D. T. W. Lui , C. H. Lee , Y. H. Chan , et al., “HbA1c Variability, in Addition to Mean HbA1c, Predicts Incident Hip Fractures in Chinese People With Type 2 Diabetes,” Osteoporosis International 31, no. 10 (2020): 1955–1964, 10.1007/s00198-020-05395-z. 32385660

[6] B. Spaetgens , S. H. A. Brouns , A. Linkens , et al., “Associations Between Presence of Diabetes, Mortality and Fracture Type in Individuals With a Hip Fracture,” Diabetes Research and Clinical Practice 192 (2022): 110084, 10.1016/j.diabres.2022.110084. 36122868

[7] T. C. Lee , Y. L. Lee , J. C. Chen , C. H. Chen , and P. S. Ho , “Impact of Type 2 Diabetes on Postoperative Outcome After Hip Fracture: Nationwide Population‐Based Study in Taiwan,” BMJ Open Diabetes Research & Care 8, no. 1 (2020): e000843, 10.1136/bmjdrc-2019-000843. PMC7039587 32086279

[8] C. Tebe , D. Martinez‐Laguna , C. Carbonell‐Abella , et al., “The Association Between Type 2 Diabetes Mellitus, Hip Fracture, and Post‐Hip Fracture Mortality: A Multi‐State Cohort Analysis,” Osteoporosis International 30, no. 12 (2019): 2407–2415, 10.1007/s00198-019-05122-3. 31444526

[9] A. Yang , H. Wu , E. S. H. Lau , et al., “Glucose‐Lowering Drug Use, Glycemic Outcomes, and Severe Hypoglycemia: 18‐Year Trends in 0.9 Million Adults With Diabetes in Hong Kong (2002‐2019),” Lancet Regional Health – Western Pacific 26 (2022): 100509, 10.1016/j.lanwpc.2022.100509. 35789825 PMC9249907

[10] R. Viggers , J. Starup‐Linde , and P. Vestergaard , “Discrepancies in Type of First Major Osteoporotic Fracture and Anti‐Osteoporotic Therapy in Elderly People With Type 2 Diabetes Mellitus: A Retrospective Danish Cohort Study,” Bone 171 (2023): 116745, 10.1016/j.bone.2023.116745. 36965654

[11] B. J. Ross , O. C. Lee , M. B. Harris , T. C. Dowd , F. H. Savoie , and W. F. Sherman , “The Impact of Diabetes on Osteoporosis Management and Secondary Fracture Risk After Primary Fragility Fractures: A Propensity Score‐Matched Cohort Study,” Journal of the American Academy of Orthopaedic Surgeons 30, no. 2 (2022): e204–e212, 10.5435/JAAOS-D-21-00185. 34543247

[12] M. S. LeBoff , S. L. Greenspan , K. L. Insogna , et al., “Correction to: The Clinician's Guide to Prevention and Treatment of Osteoporosis,” Osteoporosis International 33, no. 10 (2022): 2243, 10.1007/s00198-022-06479-8. 35900384 PMC9546943

[13] D. T. W. Lui , C. H. Lee , W. S. Chow , et al., “A Territory‐Wide Study on the Impact of COVID‐19 on Diabetes‐Related Acute Care,” Journal of Diabetes Investigation 11, no. 5 (2020): 1303–1306, 10.1111/jdi.13368. 32779868 PMC7404850

[14] D. T. W. Lui , E. H. M. Tang , I. C. H. Au , et al., “Evaluation of Fracture Risk Among Patients With Type 2 Diabetes and Nonvalvular Atrial Fibrillation Receiving Different Oral Anticoagulants,” Diabetes Care 45, no. 11 (2022): 2620–2627, 10.2337/dc22-0664. 36126158

[15] D. T. W. Lui , L. Li , X. Liu , et al., “The Association of HDL‐Cholesterol Levels With Incident Major Adverse Cardiovascular Events and Mortality in 0.6 Million Individuals With Type 2 Diabetes: A Population‐Based Retrospective Cohort Study,” BMC Medicine 22, no. 1 (2024): 586, 10.1186/s12916-024-03810-4. 39696353 PMC11657474

[16] S. Khandelwal and N. E. Lane , “Osteoporosis: Review of Etiology, Mechanisms, and Approach to Management in the Aging Population,” Endocrinology and Metabolism Clinics of North America 52, no. 2 (2023): 259–275, 10.1016/j.ecl.2022.10.009. 36948779

[17] D. T. W. Lui , T. Wu , E. H. M. Tang , et al., “Fracture Risks Associated With Sodium‐Glucose Cotransporter‐2 Inhibitors in Type 2 Diabetes Patients Across eGFR and Albuminuria Categories: A Population‐Based Study in Hong Kong,” Diabetes Research and Clinical Practice 197 (2023): 110576, 10.1016/j.diabres.2023.110576. 36780955

[18] D. T. W. Lui , I. C. H. Au , E. H. M. Tang , et al., “Kidney Outcomes Associated With Sodium‐Glucose Cotransporter 2 Inhibitors Versus Glucagon‐Like Peptide 1 Receptor Agonists: A Real‐World Population‐Based Analysis,” EClinicalMedicine 50 (2022): 101510, 10.1016/j.eclinm.2022.101510. 35784442 PMC9241106

[19] H. Johansson , K. Siggeirsdottir , N. C. Harvey , et al., “Imminent Risk of Fracture After Fracture,” Osteoporosis International 28, no. 3 (2017): 775–780, 10.1007/s00198-016-3868-0. 28028554 PMC5338733

[20] HACW , “Hospital Authority Corporate Website,” accessed 28 July 2025, https://www.ha.org.hk/visitor/ha_visitor_index.asp?Content_ID=10045&Lang=ENG&Dimension=100.

[21] E. L. Goh , A. Khatri , A. B. Costa , et al., “Prevalence of Complications in Older Adults After Hip Fracture Surgery : A Systematic Review and Meta‐Analysis,” Bone & Joint Journal 107‐B, no. 2 (2025): 139–148, 10.1302/0301-620X.107B2.BJJ-2024-0251.R1. 39889748

[22] C. C. Liao , C. S. Lin , C. C. Shih , et al., “Erratum. Increased Risk of Fracture and Postfracture Adverse Events in Patients With Diabetes: Two Nationwide Population‐Based Retrospective Cohort Studies. Diabetes Care 2014;37:2246–2252,” Diabetes Care 40, no. 8 (2017): 1134, 10.2337/dc17-er08c. PMC5521979 28615240

[23] G. E. Umpierrez , S. D. Isaacs , N. Bazargan , X. You , L. M. Thaler , and A. E. Kitabchi , “Hyperglycemia: An Independent Marker of In‐Hospital Mortality in Patients With Undiagnosed Diabetes,” Journal of Clinical Endocrinology and Metabolism 87, no. 3 (2002): 978–982, 10.1210/jcem.87.3.8341. 11889147

[24] J. A. Flores‐Le Roux , J. Comin , J. Pedro‐Botet , et al., “Seven‐Year Mortality in Heart Failure Patients With Undiagnosed Diabetes: An Observational Study,” Cardiovascular Diabetology 10 (2011): 39, 10.1186/1475-2840-10-39. 21569580 PMC3125195

[25] R. R. Giraldez , R. M. Clare , R. D. Lopes , et al., “Prevalence and Clinical Outcomes of Undiagnosed Diabetes Mellitus and Prediabetes Among Patients With High‐Risk Non‐ST‐Segment Elevation Acute Coronary Syndrome,” American Heart Journal 165, no. 6 (2013): 918–925.e2, 10.1016/j.ahj.2013.01.005. 23708162

[26] P. R. Ebeling , H. H. Nguyen , J. Aleksova , A. J. Vincent , P. Wong , and F. Milat , “Secondary Osteoporosis,” Endocrine Reviews 43, no. 2 (2022): 240–313, 10.1210/endrev/bnab028. 34476488

[27] Y. Chen , X. Yang , K. Meng , et al., “Stress‐Induced Hyperglycemia After Hip Fracture and the Increased Risk of Acute Myocardial Infarction in Nondiabetic Patients,” Diabetes Care 36, no. 10 (2013): 3328–3332, 10.2337/dc13-0119. 23846813 PMC3781495

[28] D. K. H. Yee , T. W. Lau , C. Fang , K. Ching , J. Cheung , and F. Leung , “Orthogeriatric Multidisciplinary co‐Management Across Acute and Rehabilitation Care Improves Length of Stay, Functional Outcomes and Complications in Geriatric Hip Fracture Patients,” Geriatric Orthopaedic Surgery & Rehabilitation 13 (2022): 21514593221085813, 10.1177/21514593221085813. 35433103 PMC9006372

[29] C. Poiana and C. Capatina , “Fracture Risk Assessment in Patients With Diabetes Mellitus,” Journal of Clinical Densitometry 20, no. 3 (2017): 432–443, 10.1016/j.jocd.2017.06.011. 28716499

[30] D. Vinther , R. W. Thomsen , O. Furnes , J. E. Gjertsen , and A. B. Pedersen , “Impact of Diabetes on the Risk of Subsequent Fractures in 92,600 Patients With an Incident Hip Fracture: A Danish Nationwide Cohort Study 2004‐2018,” Bone 184 (2024): 117104, 10.1016/j.bone.2024.117104. 38636621

