# Supplementary material for: Type 2 Diabetes Is Associated With Increased Complications and Mortality After Hip Fracture in Older Adults Aged 60 Years or Older
Source: Diabetes Obes Metab. 2026 Jun 16;28(9):8084–93. doi: 10.1111/dom.71000 (PMC13448971; doi:10.1111/dom.71000)
Supplement: Supplementary file 1 [file DOM-28-8084-s001.docx]

**Supplementary Figure 1.**	Study flow chart

**Supplementary Table 1.** ICD-9-CM Codes for identification of components in the modified Charlson comorbidity index

| **Comorbidities** | **ICD-9 CM Code** |
| --- | --- |
| Myocardial infarction | 410 |
| Peripheral vascular disease | 443.9, 441.x, 785.4, V43.4 |
| Cerebrovascular disease | 430-438 |
| Dementia | 290, 291.1x, 294.2x, 331.0x,331.1x, 331.82 |
| chronic obstructive pulmonary disease | 490-496, 500-505, 506.4 |
| Rheumatoid arthritis and other inflammatory polyarthropathies | 710.0, 710.1, 710.4, 714.0-714.2, 714.81, 725 |
| Ulcers | 531-534 |
| Mild liver disease | 571.2, 571.4-571.6 |
| Moderate or severe liver disease | 456.0-456.2,572.2-572.4, 572.8 |
| Hemiplegia or paraplegia | 342, 344.1 |
| Renal disease | 582, 585-586, 588, 583.0-583.2, 583.4, 583.6-583.7 |
| Any malignancy | 140-159, 160-165, 170-172, 174-176, 179, 180-189, 190-195, 200-208 |
| Metastatic solid tumour | 196-199 |
| AIDS/HIV | 042-044 |

**Supplementary Table 2.** Risks of in‑hospital complications after hip fracture among patients without and with pre‑existing diabetes, where patients with pre-existing diabetes were stratified by the number of classes of anti‑diabetic medications used

|  | **Number of patients** | **Events** | **Incidence**  **(%)** | **Adjusted OR**  **(95% CI)** | **p** |  |  |  |
| --- | --- | --- | --- | --- | --- | --- | --- | --- |
| **Any in-hospital complications** |  |  |  |  |  |  |  |  |
| No diabetes | 34342 | 5146 | 15.0% | Reference | | |  |  |
| Pre-existing diabetes: number of classes of anti-diabetic medication |  |  |  |  |  |  |  |  |
| 0 | 9695 | 1630 | 16.8% | 1.09 (1.03-1.16) | 0.01 |  |  |  |
| 1 | 4909 | 857 | 17.5% | 1.17 (1.08-1.27) | <0.001 |  |  |  |
| ≥2 | 3526 | 611 | 17.3% | 1.28 (1.16-1.4) | <0.001 |  |  |  |
| **Infective complications** |  |  |  |  |  |  |  |  |
| No diabetes | 34342 | 3245 | 9.4% | Reference | | | | |
| Pre-existing diabetes: number of classes of anti-diabetic medication |  |  |  |  |  |  |  |  |
| 0 | 9695 | 957 | 9.9% | 1.04 (0.96-1.12) | 0.31 |  |  |  |
| 1 | 4909 | 507 | 10.3% | 1.13 (1.02-1.25) | 0.02 |  |  |  |
| ≥2 | 3526 | 366 | 10.4% | 1.21 (1.07-1.35) | 0.001 |  |  |  |
| **Septicaemia** |  |  |  |  |  |  |  |  |
| No diabetes | 34342 | 563 | 1.6% | Reference | | | |  |
| Pre-existing diabetes: number of classes of anti-diabetic medication |  |  |  |  |  |  |  |  |
| 0 | 9695 | 198 | 2.0% | 1.22 (1.04-1.44) | 0.02 |  |  |  |
| 1 | 4909 | 90 | 1.8% | 1.11 (0.89-1.39) | 0.36 |  |  |  |
| ≥2 | 3526 | 76 | 2.2% | 1.38 (1.08-1.77) | 0.01 |  |  |  |
| **Acute renal failure** |  |  |  |  |  |  |  |  |
| No diabetes | 34342 | 185 | 0.5% | Reference | | | |  |
| Pre-existing diabetes: number of classes of anti-diabetic medication |  |  |  |  |  |  |  |  |
| 0 | 9695 | 101 | 1.0% | 1.85 (1.45-2.37) | <0.001 |  |  |  |
| 1 | 4909 | 46 | 0.9% | 1.74 (1.26-2.42) | <0.001 |  |  |  |
| ≥2 | 3526 | 46 | 1.3% | 2.68 (1.93-3.73) | <0.001 |  |  |  |
| **Acute ischaemic stroke** |  |  |  |  |  |  |  |  |
| No diabetes | 34342 | 1725 | 5.0% | Reference | | | | |
| Pre-existing diabetes: number of classes of anti-diabetic medication |  |  |  |  |  |  |  |  |
| 0 | 9695 | 628 | 6.5% | 1.16 (1.05-1.28) | 0.003 |  |  |  |
| 1 | 4909 | 312 | 6.4% | 1.13 (1-1.29) | 0.06 |  |  |  |
| ≥2 | 3526 | 228 | 6.5% | 1.33 (1.15-1.54) | <0.001 |  |  |  |
| **Acute myocardial infarction** |  |  |  |  |  |  |  |  |
| No diabetes | 34342 | 416 | 1.2% | Reference | | | |  |
| Pre-existing diabetes: number of classes of anti-diabetic medication |  |  |  |  |  |  |  |  |
| 0 | 9695 | 162 | 1.7% | 1.31 (1.09-1.58) | 0.004 |  |  |  |
| 1 | 4909 | 88 | 1.8% | 1.48 (1.17-1.87) | <0.001 |  |  |  |
| ≥2 | 3526 | 44 | 1.2% | 1.14 (0.83-1.56) | 0.42 |  |  |  |
| **Acute pulmonary embolism** |  |  |  |  |  |  |  |  |
| No diabetes | 34342 | 134 | 0.4% | Reference | | | |  |
| Pre-existing diabetes: number of classes of anti-diabetic medication |  |  |  |  |  |  |  |  |
| 0 | 9695 | 37 | 0.4% | 0.99 (0.69-1.44) | 0.98 |  |  |  |
| 1 | 4909 | 18 | 0.4% | 0.97 (0.59-1.59) | 0.89 |  |  |  |
| ≥2 | 3526 | 9 | 0.3% | 0.67 (0.34-1.32) | 0.24 |  |  |  |

Abbreviations: OR, odds ratio; CI, confidence interval

Adjusted for sex, age, obesity, modified Charlson comorbidity index, and year of admission

**Supplementary Table 3.** Independent factors associated with post-hip fractures outcomes among patients without and with pre‑existing diabetes, where patients with pre-existing diabetes were stratified by the number of classes of anti‑diabetic medications used

|  | **Crude HR (95% CI)** | | **p** | | **Adjusted HR (95% CI)** | | **p** | |  |  |
| --- | --- | --- | --- | --- | --- | --- | --- | --- | --- | --- |
| **Prolonged hospitalisation** |  | |  | |  | |  | |  |  |
| No diabetes | Reference | | | | Reference | | | | |  |
| Pre-existing diabetes: number of classes of anti-diabetic medication |  | |  | |  | |  | | |  |
| 0 | 0.98 (0.95-1.01) | | 0.13 | | 0.98 (0.95-1.01) | | 0.15* | | |  |
| 1 | 1.03 (0.99-1.07) | | 0.23 | | 1.02 (0.98-1.06) | | 0.36* | | |  |
| ≥2 | 0.98 (0.94-1.03) | | 0.50 | | 0.97 (0.93-1.02) | | 0.19* | | |  |
| **In-hospital mortality** | |  | |  | |  | |  | | |
| No diabetes | Reference | | | | Reference | | | | |  |
| Pre-existing diabetes: number of classes of anti-diabetic medication |  | |  | |  | |  | | |  |
| 0 | 0.95 (0.83-1.08) | | 0.41 | | 0.92 (0.81-1.05) | | 0.21* | | |  |
| 1 | 0.89 (0.74-1.06) | | 0.17 | | 0.91 (0.77-1.09) | | 0.32* | | |  |
| ≥2 | 0.85 (0.70-1.04) | | 0.12 | | 0.95 (0.78-1.17) | | 0.63* | | |  |
| **1-year mortality** |  | |  | |  | |  | | |  |
| No diabetes | Reference | | | | Reference | | | | |  |
| Pre-existing diabetes: number of classes of anti-diabetic medication |  | |  | |  | |  | | |  |
| 0 | 1.18 (1.12-1.25) | | <0.001 | | 1.10 (1.04-1.16) | | <0.001* | | |  |
| 1 | 1.03 (0.96-1.11) | | 0.38 | | 1.01 (0.94-1.09) | | 0.70* | | |  |
| ≥2 | 0.92 (0.85-1.01) | | 0.08 | | 1.03 (0.94-1.13) | | 0.50* | | |  |
| **Initiation of osteoporosis treatment in 2 years**  **(n=50097)** |  | |  | |  | |  | | |  |
| No diabetes |  | |  | |  | |  | | |  |
| Pre-existing diabetes: number of classes of anti-diabetic medication | Reference | | | | Reference | | | | |  |
| 0 | 0.99 (0.94-1.04) | | 0.74 | | 0.99 (0.94-1.04) | | 0.65^@^ | | |  |
| 1 | 1.01 (0.94-1.08) | | 0.85 | | 0.97 (0.90-1.04) | | 0.35^@^ | | |  |
| ≥2 | 1.15 (1.07-1.24) | | <0.001 | | 1.00 (0.93-1.08) | | 0.92^@^ | | |  |
| **Recurrent fractures in 2 years** |  | |  | |  | |  | | |  |
| No diabetes | Reference | | | | Reference | | | | |  |
| Pre-existing diabetes: number of classes of anti-diabetic medication |  | |  | |  | |  | | |  |
| 0 | 0.97 (0.89-1.06) | | 0.48 | | 0.96 (0.87-1.05) | | 0.31^#^ | | |  |
| 1 | 1.01 (0.90-1.14) | | 0.85 | | 1.00 (0.89-1.12) | | 0.96^#^ | | |  |
| ≥2 | 1.15 (1.02-1.31) | | 0.03 | | 1.14 (1.00-1.30) | | 0.05^#^ | | |  |

Abbreviations: HR, hazards ratio; CI, confidence interval

*adjusted for sex, age, obesity, modified Charlson comorbidity index, and year of admission

^@^ adjusted for sex, age, obesity, modified Charlson comorbidity index, year of admission, and history of major osteoporotic fractures

^#^ adjusted for sex, age, obesity, modified Charlson comorbidity index, year of admission, history of osteoporosis treatment and history of major osteoporotic fractures

**Supplementary Table 4.** Risks of various complications during hospitalization for hip fractures among non-diabetes, pre-existing diabetes and undiagnosed diabetes

|  | **Number of patients** | **Events** | **Incidence**  **(%)** | **Adjusted OR**  **(95% CI)** | **p** | **Adjusted OR**  **(95% CI)** | **p** |
| --- | --- | --- | --- | --- | --- | --- | --- |
| **Any in-hospital complications** |  |  |  |  |  |  |  |
| No diabetes | 34132 | 5098 | 14.9% | Reference | |  |  |
| Pre-existing diabetes | 18130 | 3096 | 17.1% | 1.18 (1.12-1.24) | <0.001 | Reference | |
| Undiagnosed diabetes | 210 | 50 | 23.0% | 2.49 (1.79-3.47) | <0.001 | 2.15 (1.54-3.00) | <0.001 |
| **Infective complications** |  |  |  |  |  |  |  |
| No diabetes | 34132 | 3215 | 9.4% | Reference | |  |  |
| Pre-existing diabetes | 18130 | 1829 | 10.1% | 1.11 (1.04-1.18) | <0.001 | Reference | |
| Undiagnosed diabetes | 210 | 31 | 14.3% | 1.91 (1.29-2.82) | 0.001 | 1.72 (1.16-2.55) | 0.01 |
| **Septicaemia** |  |  |  |  |  |  |  |
| No diabetes | 34132 | 554 | 1.6% | Reference | |  |  |
| Pre-existing diabetes | 18130 | 363 | 2.0% | 1.25 (1.09-1.44) | 0.001 | Reference | |
| Undiagnosed diabetes | 210 | 10 | 4.6% | 3.40 (1.73-6.69) | <0.001 | 2.63 (1.33-5.21) | 0.01 |
| **Acute renal failure** |  |  |  |  |  |  |  |
| No diabetes | 34132 | 182 | 0.5% | Reference | |  |  |
| Pre-existing diabetes | 18130 | 193 | 1.1% | 2.00 (1.63-2.46) | <0.001 | Reference | |
| Undiagnosed diabetes | 210 | 3 | 1.4% | 3.16 (1.00-10.03) | 0.051 | 1.47 (0.46-4.66) | 0.52 |
| **Acute ischaemic stroke** |  |  |  |  |  |  |  |
| No diabetes | 34132 | 1716 | 5.0% | Reference | |  |  |
| Pre-existing diabetes | 18130 | 1167 | 6.4% | 1.25 (1.15-1.35) | <0.001 | Reference | |
| Undiagnosed diabetes | 210 | 10 | 4.6% | 1.91 (0.96-3.81) | 0.07 | 1.54 (0.77-3.07) | 0.22 |
| **Acute myocardial infarction** |  |  |  |  |  |  |  |
| No diabetes | 34132 | 400 | 1.2% | Reference | |  |  |
| Pre-existing diabetes | 18130 | 294 | 1.6% | 1.40 (1.20-1.63) | <0.001 | Reference | |
| Undiagnosed diabetes | 210 | 16 | 7.4% | 8.09 (4.77-13.72) | <0.001 | 6.10 (3.56-10.43) | <0.001 |
| **Acute pulmonary embolism** |  |  |  |  |  |  |  |
| No diabetes | 34132 | 133 | 0.4% | Reference | |  |  |
| Pre-existing diabetes | 18130 | 63 | 0.3% | 0.92 (0.68-1.24) | 0.57 | Reference | |
| Undiagnosed diabetes | 210 | 2 | 0.9% | 1.09 (0.15-7.89) | 0.93 | 1.16 (0.16-8.46) | 0.89 |

Abbreviations: OR, odds ratio; CI, confidence interval

Adjusted for sex, age, obesity, modified Charlson comorbidity index, and year of admission

**Supplementary Table 5.** Independent factors associated with post-hip fractures outcomes among non-diabetes, pre-existing diabetes and undiagnosed diabetes

|  | **Crude HR (95% CI)** | **p** | **Adjusted HR (95% CI)** | **p** | **Adjusted HR (95% CI)** | | | | | **p** | | | | |
| --- | --- | --- | --- | --- | --- | --- | --- | --- | --- | --- | --- | --- | --- | --- |
| **Prolonged hospitalization** |  |  |  |  |  | | | | |  | | | | |
| No diabetes | Reference | | Reference | |  | | | | | | | | | |
| Pre-existing diabetes | 0.99 (0.96-1.01) | 0.35 | 0.99 (0.96-1.01) | 0.24* | Reference | | | | | | | | | |
| Undiagnosed diabetes | 0.77 (0.66-0.90) | <0.001 | 0.74 (0.63-0.86) | <0.001* | 0.75 (0.64-0.87) | | | | | <0.001* | | | | |
| **In-hospital mortality** |  |  |  |  |  | | | | |  | | | | |
| No diabetes | Reference | | Reference | |  | | |  | | | | | | |
| Pre-existing diabetes | 0.92 (0.83-1.02) | 0.10 | 0.94 (0.84-1.04) | 0.23* | Reference | | | | | | | | | |
| Undiagnosed diabetes | 1.47 (0.97-2.49) | 0.15 | 1.86 (1.10-3.17) | 0.02* | 1.94 (1.14-3.33) | | | | | 0.02* | | | | |
| **1-year mortality** |  |  |  |  |  | | | | |  | | | | |
| No diabetes | Reference | | Reference | |  | | |  | | | | | | |
| Pre-existing diabetes | 1.09 (1.05-1.14) | <0.001 | 1.07 (1.02-1.11) | 0.004* | Reference | | | | | | | | | |
| Undiagnosed diabetes | 1.17 (0.85-1.60) | 0.33 | 1.46 (1.07-2.01) | 0.02* | 1.35 (0.99-1.86) | | | | | 0.06* | | | | |
| **Initiation of osteoporosis treatment in 2 years** | | | | | | | | | | |  |  |  |  |
| No diabetes | Reference | | Reference | | |  | | |  | | | | |  |
| Pre-existing diabetes | 1.01 (0.97-1.06) | 0.21 | 0.98 (0.94-1.03) | 0.46^@^ | | Reference | | | | | | | |  |
| Undiagnosed diabetes | 1.08 (0.79-1.48) | 0.61 | 0.81 (0.59-1.11) | 0.19^@^ | | 0.83 (0.60-1.14) | | | | 0.24^@^ | | | | |
| **Recurrent fractures in 2 years** | | | | | | | | | | | | |  |  |
| No diabetes | Reference | | Reference | | |  |  | | | | |  |  |  |
| Pre-existing diabetes | 1.02 (0.95-1.09) | 0.64 | 1.00 (0.93-1.08) | 0.95^#^ | | Reference | | | | | |  |  |  |
| Undiagnosed diabetes | 1.09 (0.66-1.82) | 0.73 | 1.09 (0.66-1.81) | 0.74^#^ | | 1.03 (0.62-1.71) | | | | 0.92^#^ | | | | |

Abbreviations: HR, hazards ratio; CI, confidence interval

*adjusted for sex, age, obesity, modified Charlson comorbidity index, and year of admission

^@^adjusted for sex, age, obesity, modified Charlson comorbidity index, year of admission, and history of major osteoporotic fractures

^#^adjusted for sex, age, obesity, modified Charlson comorbidity index, year of admission, history of osteoporosis treatment and history of major osteoporotic fractures

**Supplementary Table 6** Risks of various complications during hospitalization for hip fractures among non-diabetes, pre-existing diabetes, pre-existing undiagnosed diabetes, and fracture induced stress hyperglycaemia

|  | **Number of patients** | **Events** | **Incidence**  **(%)** | **Adjusted OR**  **(95% CI)** | | **p** | | **Adjusted OR**  **(95% CI)** | | **p** | |
| --- | --- | --- | --- | --- | --- | --- | --- | --- | --- | --- | --- |
| **Any in-hospital complications** |  |  |  |  | |  | |  | |  | |
| No diabetes | 34132 | 5098 | 14.9% | Reference | | |  | |  | |  |
| Pre-existing diabetes | 18130 | 3096 | 17.1% | 1.18 (1.12-1.24) | | <0.001 | | Reference | | | |
| Pre-existing unrecognised diabetes | 82 | 13 | 15.9% | 1.40 (0.76-2.56) | | 0.28 | | 1.19 (0.65-2.17) | | 0.58 | |
| Fracture induced stress hyperglycaemia | 121 | 34 | 28.1% | 3.51 (2.34-5.27) | | <0.001 | | 3.09 (2.05-4.64) | | <0.001 | |
| **Infective complications** |  |  |  |  | |  | |  | |  | |
| No diabetes | 34132 | 3215 | 9.4% | Reference | | |  | |  | |  |
| Pre-existing diabetes | 18130 | 1829 | 10.1% | 1.11 (1.04-1.18) | | <0.001 | Reference | | | |  |
| Pre-existing unrecognised diabetes | 82 | 11 | 13.4% | 1.72 (0.91-3.27) | | 0.10 | | 1.53 (0.81-2.91) | | 0.19 | |
| Fracture induced stress hyperglycaemia | 121 | 18 | 14.9% | 2.02 (1.22-3.34) | | 0.01 | | 1.84 (1.1-3.05) | | 0.02 | |
| **Septicaemia** |  |  |  |  | |  | |  | |  | |
| No diabetes | 34132 | 554 | 1.6% | Reference | | |  | |  | |  |
| Pre-existing diabetes | 18130 | 363 | 2.0% | 1.25 (1.09-1.44) | | 0.001 | Reference | | | |  |
| Pre-existing unrecognised diabetes | 82 | 3 | 3.7% | 2.68 (0.84-8.55) | | 0.10 | | 2.09 (0.65-6.67) | | 0.22 | |
| Fracture induced stress hyperglycaemia | 121 | 6 | 5.0% | 4.12 (1.8-9.47) | | <0.001 | | 3.2 (1.38-7.4) | | 0.01 | |
| **Acute renal failure** |  |  |  |  | |  | |  | |  | |
| No diabetes | 34132 | 182 | 0.5% | Reference | | |  | |  | |  |
| Pre-existing diabetes | 18130 | 193 | 1.1% | 2.00 (1.63-2.46) | | <0.001 | Reference | | | |  |
| Pre-existing unrecognised diabetes | 82 | 1 | 1.2% | 2.87 (0.4-20.81) | | 0.30 | | 1.35 (0.19-9.81) | | 0.77 | |
| Fracture induced stress hyperglycaemia | 121 | 2 | 1.7% | 3.49 (0.85-14.34) | | 0.08 | | 1.61 (0.39-6.63) | | 0.51 | |
| **Acute ischaemic stroke** |  |  |  |  | |  | |  | |  | |
| No diabetes | 34132 | 1716 | 5.0% | Reference | | |  | |  | |  |
| Pre-existing diabetes | 18130 | 1167 | 6.4% | 1.25 (1.15-1.35) | | <0.001 | Reference | | | |  |
| Pre-existing unrecognised diabetes | 82 | 2 | 2.4% | 0.79 (0.19-3.29) | | 0.74 | | 0.63 (0.15-2.61) | | 0.52 | |
| Fracture induced stress hyperglycaemia | 121 | 7 | 5.8% | 3.25 (1.48-7.14) | | 0.001 | | 2.67 (1.21-5.89) | | 0.02 | |
| **Acute myocardial infarction** |  |  |  |  | |  | |  | |  | |
| No diabetes | 34132 | 400 | 1.2% | Reference | | |  | |  | |  |
| Pre-existing diabetes | 18130 | 294 | 1.6% | 1.40 (1.20-1.63) | <0.001 | | Reference | | | |  |
| Pre-existing unrecognised diabetes | 82 | 4 | 4.9% | 5.38 (1.94-14.87) | | 0.001 | | 4.04 (1.45-11.23) | | 0.01 | |
| Fracture induced stress hyperglycaemia | 95 | 12 | 9.9% | 10.21 (5.51-18.91) | | <0.001 | | 7.73 (4.13-14.49) | | <0.001 | |
| **Acute pulmonary embolism** |  |  |  |  | |  | |  | |  | |
| No diabetes | 34132 | 133 | 0.4% | Reference | | |  | |  | |  |
| Pre-existing diabetes | 18130 | 63 | 0.3% | 0.92 (0.68-1.24) | 0.57 | | Reference | | | |  |
| Pre-existing unrecognised diabetes | 82 | 0 | 0.00% | - | | - | | - | | - | |
| Fracture induced stress hyperglycaemia | 121 | 1 | 0.8% | 1.80 (0.25-13.09) | | 0.56 | | 1.92 (0.26-14.25) | | 0.52 | |

Abbreviations: OR, odds ratio; CI, confidence interval

Adjusted for sex, age, obesity, modified Charlson comorbidity index, and year of admission

**Supplementary Table 7.** Independent factors associated with post-hip fractures outcomes among non-diabetes, pre-existing diabetes, pre-existing undiagnosed diabetes, and fracture induced stress hyperglycaemia

|  | **Crude HR (95% CI)** | **p** | **Adjusted HR (95% CI)** | **p** | **Adjusted HR (95% CI)** | | | **p** | |  |  |  |  |
| --- | --- | --- | --- | --- | --- | --- | --- | --- | --- | --- | --- | --- | --- |
| **Prolonged hospitalization** |  |  |  |  |  | | |  | |  |  |  |  |
| No diabetes | Reference | | Reference | | |  |  | | | | | |  |
| Pre-existing diabetes | 0.99 (0.96-1.01) | 0.35 | 0.99 (0.96-1.01) | 0.24* | Reference | | | | |  |  |  |  |
| Pre-existing unrecognised diabetes | 0.90 (0.69-1.18) | 0.44 | 0.84 (0.64-1.09) | 0.19* | 0.90 (0.37 – 2.17) | | | 0.81* | |  |  |  |  |
| Fracture induced stress hyperglycaemia | 0.70 (0.58-0.83) | <0.001 | 0.67 (0.56-0.80) | <0.001* | 1.17 (0.63 – 2.18) | | | 0.61* | |  |  |  |  |
| **In-hospital mortality** |  |  |  |  |  | | |  | |  |  |  |  |
| No diabetes | Reference | | Reference | | |  |  | | | | | |  |
| Pre-existing diabetes | 0.92 (0.83-1.02) | 0.10 | 0.94 (0.84-1.04) | 0.23* | Reference | | | | |  |  |  |  |
| Pre-existing unrecognised diabetes | 1.89 (0.90-3.97) | 0.09 | 0.95 (0.39-2.30) | 0.91* | 2.92 (1.38 – 6.21) | | | 0.01* | |  |  |  |  |
| Fracture induced stress hyperglycaemia | 1.25 (0.60-2.63) | 0.55 | 1.48 (0.70-3.12) | 0.31* | 1.49 (0.70 – 3.17) | | | 0.30* | |  |  |  |  |
| **1-year mortality** |  |  |  |  |  | | |  | |  |  |  |  |
| No diabetes | Reference | | Reference | | |  | | | | | | |  |
| Pre-existing diabetes | 1.09 (1.05-1.14) | <0.001 | 1.07 (1.02-1.11) | 0.004* | Reference | | | | |  |  |  |  |
| Pre-existing unrecognised diabetes | 1.31 (0.81-2.10) | 0.92 | 1.85 (1.15-2.98) | 0.01* | 1.71 (1.06 – 2.76) | | | 0.03* | |  |  |  |  |
| Fracture induced stress hyperglycaemia | 1.15 (0.76-1.75) | 0.51 | 1.33 (0.87-2.03) | 0.18* | 1.21 (0.80 – 1.85) | | | 0.37* | |  |  |  |  |
| **Initiation of osteoporosis treatment in 2 years** |  |  |  |  |  | | |  | | | |  |  |
| No diabetes | Reference | | Reference | | |  | | |  | | | | |
| Pre-existing diabetes | 1.01 (0.97-1.06) | 0.21 | 0.98 (0.94-1.03) | 0.46^@^ | Reference | | | | | |  |  |  |
| Pre-existing unrecognised diabetes | 0.56 (0.29-1.09) | 0.09 | 0.46 (0.24-0.90) | 0.02^@^ | 0.85 (0.65 – 1.12) | | | 0.25^@^ | | | |  |  |
| Fracture induced stress hyperglycaemia | 1.45 (1.00-2.11) | 0.05 | 1.04 (0.72-1.51) | 0.84^@^ | 0.68 (0.56 – 0.82) | | | <0.001^@^ | | | |  |  |
| **Recurrent fractures in 2 years** |  |  |  |  |  | | |  | | | |  |  |
| No diabetes | Reference | | Reference | | |  | | |  | | | | |
| Pre-existing diabetes | 1.02 (0.95-1.09) | 0.64 | 1.00 (0.93-1.08) |  | Reference | | | | | |  |  |  |
| Pre-existing unrecognised diabetes | 0.93 (0.39-2.25) | 0.88 | 0.95 (0.39-2.30) | 0.91^#^ | 0.90 (0.37 – 2.17) | | | 0.81^#^ | | | |  |  |
| Fracture induced stress hyperglycaemia | 1.27 (0.68-2.35) | 0.45 | 1.30 (0.70-2.41) | 0.40^#^ | 1.17 (0.63 – 2.18) | | | 0.61^#^ | | | |  |  |

Abbreviations: HR, hazards ratio; CI, confidence interval

*adjusted for sex, age, obesity, modified Charlson comorbidity index, and year of admission

^@^ adjusted for sex, age, obesity, modified Charlson comorbidity index, year of admission, and history of major osteoporotic fractures

^#^ adjusted for sex, age, obesity, modified Charlson comorbidity index, year of admission, history of osteoporosis treatment and history of major osteoporotic fractures

**Supplementary Table 8.** Risks of various complications during hospitalization for hip fractures (transcervical type; ICD-9 820.0-1) among patients with and without diabetes

|  | **Number of patients** | **Events** | **Incidence** | **Adjusted OR*** | **95% CI** | **P value** | | |
| --- | --- | --- | --- | --- | --- | --- | --- | --- |
| **Any in-hospital complications** |  |  |  |  |  |  | | |
| No diabetes | 16330 | 2329 | 14.3% | Reference | | | | |
| Diabetes | 8536 | 1437 | 16.8% | 1.19 | 1.11-1.28 | <0.001 | |  |
| **Infective complications** |  |  |  |  |  |  | |  |
| No diabetes | 16330 | 1487 | 9.1% | Reference | | | |  |
| Diabetes | 8536 | 859 | 10.1% | 1.14 | 1.04-1.24 | 0.01 | |  |
| **Septicaemia** |  |  |  |  |  |  | | |
| No diabetes | 16330 | 253 | 1.5% | Reference | | | | |
| Diabetes | 8536 | 178 | 2.1% | 1.37 | 1.12-1.66 | 0.002 |  |  |
| **Acute renal failure** |  |  |  |  |  |  | | |
| No diabetes | 16330 | 68 | 0.4% | Reference | | | | |
| Diabetes | 8536 | 90 | 1.1% | 2.47 | 1.79-3.4 | <0.001 | | |
| **Acute ischaemic stroke** |  |  |  |  |  |  | | |
| No diabetes | 16330 | 797 | 4.9% | Reference | | | | |
| Diabetes | 8536 | 554 | 6.5% | 1.22 | 1.09-1.38 | <0.001 | | |
| **Acute myocardial infarction** |  |  |  |  |  |  | | |
| No diabetes | 16330 | 163 | 1.0% | Reference | | | | |
| Diabetes | 8536 | 102 | 1.2% | 1.16 | 0.90-1.50 | 0.24 | | |
| **Acute pulmonary embolism** |  |  |  |  |  |  | | |
| No diabetes | 16330 | 50 | 0.3% | Reference | | | | |
| Diabetes | 8536 | 31 | 0.4% | 1.19 | 0.75-1.87 | 0.46 | | |

Abbreviations: OR, odds ratio; CI, confidence interval

*Adjusted for sex, age, obesity, modified Charlson comorbidity index, and year of admission

**Supplementary Table 9.** Independent association between diabetes and various post-hip fractures outcomes among patients with hip fractures (transcervical type; ICD-9 820.0-1)

|  | **Crude HR (95% CI)** | **p** | **Adjusted HR (95% CI)** | **p** |
| --- | --- | --- | --- | --- |
| Prolonged hospitalization | 0.99 (0.95-1.02) | 0.40 | 0.98 (0.95-1.02) | 0.30* |
| Mortality during hospitalization | 1.07 (0.91-1.26) | 0.42 | 1.08 (0.92-1.28) | 0.35* |
| Mortality within 1 year | 1.17 (1.09-1.25) | <0.001 | 1.11 (1.04-1.19) | 0.003* |
| Initiation of anti-osteoporosis treatment in 2 years (n=24011) | 0.98 (0.92-1.03) | 0.39 | 0.94 (0.89-1.00) | 0.05^@^ |
| Recurrent fractures in 2 years | 1.12 (1.01-1.24) | 0.04 | 1.11 (1.00-1.23) | 0.06^#^ |

Abbreviations: HR, hazards ratio; CI, confidence interval

*adjusted for sex, age, obesity, modified Charlson comorbidity index, and year of admission

^@^adjusted for sex, age, obesity, modified Charlson comorbidity index, year of admission, and history of major osteoporotic fractures

^#^ adjusted for sex, age, obesity, modified Charlson comorbidity index, year of admission, history of osteoporosis treatment and history of major osteoporotic fractures

**Supplementary Table 10.** Risks of various complications during hospitalization for hip fractures (pertrochanteric type; ICD-9 820.2-3) among patients with and without diabetes

|  | **Number of patients** | **Events** | **Incidence** | **Adjusted OR*** | **95% CI** | | **P value** | | |
| --- | --- | --- | --- | --- | --- | --- | --- | --- | --- |
| **Any in-hospital complications** |  |  |  |  |  | |  | | |
| No diabetes | 676 | 187 | 14.9% | Reference | |  | |  | |
| Diabetes | 1257 | 118 | 17.5% | 1.19 | 0.92-1.54 | | 0.20 | |  |
| **Infective complications** |  |  |  |  |  | |  | |  |
| No diabetes | 676 | 116 | 9.2% |  | Reference | | | |  |
| Diabetes | 1257 | 67 | 9.9% | 1.10 | 0.79-1.51 | | 0.58 | |  |
| **Septicaemia** |  |  |  |  |  | |  | | |
| No diabetes | 676 | 26 | 2.1% | 1.00 | Reference | | | | |
| Diabetes | 1257 | 19 | 2.8% | 1.38 | 0.75-2.54 | | 0.29 | |  |
| **Acute renal failure** |  |  |  |  |  | |  | | |
| No diabetes | 676 | 7 | 0.6% | 1.00 | Reference | | | | |
| Diabetes | 1257 | 7 | 1.0% | 1.67 | 0.56-4.94 | | 0.36 | | |
| **Acute ischaemic stroke** |  |  |  |  |  | |  | | |
| No diabetes | 676 | 56 | 4.5% | 1.00 | Reference | | | | |
| Diabetes | 1257 | 48 | 7.1% | 1.53 | 1.01-2.32 | | 0.05 | | |
| **Acute myocardial infarction** |  |  |  |  |  | |  | | |
| No diabetes | 676 | 18 | 1.4% | 1.00 | Reference | | | | |
| Diabetes | 1257 | 13 | 1.9% | 1.37 | 0.66-2.84 | | 0.40 | | |
| **Acute pulmonary embolism** |  |  |  |  |  | |  | | |
| No diabetes | 676 | 11 | 0.9% | 1.00 | Reference | | | | |
| Diabetes | 1257 | 3 | 0.4% | 0.52 | 0.14-1.88 | | 0.32 | | |

Abbreviations: OR, odds ratio; CI, confidence interval

*Adjusted for sex, age, obesity, modified Charlson comorbidity index, and year of admission

**Supplementary Table 11.** Independent association between diabetes and various post-hip fractures outcomes among patients with hip fractures (pertrochanteric type; ICD-9 820.2-3)

|  | **Crude HR (95% CI)** | **p** | **Adjusted HR (95% CI)** | **p** |
| --- | --- | --- | --- | --- |
| Prolonged hospitalization | 1.05 (0.83-1.18) | 0.46 | 1.05 (0.93-1.18) | 0.46* |
| Mortality during hospitalization | 0.80 (0.48-1.34) | 0.39 | 0.99 (0.95-1.35) | 0.41* |
| Mortality within 1 year | 1.17 (1.09-1.25) | <0.001 | 0.92 (0.73-1.15) | 0.45* |
| Initiation of anti-osteoporosis treatment in 2 years (n=1774) | 1.05 (0.83-1.31) | 0.70 | 1.05 (0.83-1.32) | 0.70^@^ |
| Recurrent fractures in 2 years | 0.79 (0.52-1.20) | 0.27 | 0.81 (0.53-1.24) | 0.33^#^ |

Abbreviations: HR, hazards ratio; CI, confidence interval

*adjusted for sex, age, obesity, modified Charlson comorbidity index, and year of admission

^@^adjusted for sex, age, obesity, modified Charlson comorbidity index, year of admission, and history of major osteoporotic fractures

^#^ adjusted for sex, age, obesity, modified Charlson comorbidity index, year of admission, history of osteoporosis treatment and history of major osteoporotic fractures

**Supplementary Table 12.** Risks of various complications during hospitalization for hip fractures among patients with and without diabetes (excluding patients with a history of cancer)

|  | **Number of patients** | **Events** | **Incidence** | **Adjusted OR*** | **95% CI** | **P value** | | |
| --- | --- | --- | --- | --- | --- | --- | --- | --- |
| **Any in-hospital complications** |  |  |  |  |  |  | | |
| No diabetes | 31413 | 4665 | 14.9% | Reference | | | | |
| Diabetes | 16422 | 2846 | 17.3% | 1.17 | 1.11-1.23 | <0.001 | |  |
| **Infective complications** |  |  |  |  |  |  | |  |
| No diabetes | 31413 | 2924 | 9.3% | Reference | | | |  |
| Diabetes | 16422 | 1663 | 10.1% | 1.12 | 1.05-1.20 | <0.001 | |  |
| **Septicaemia** |  |  |  |  |  |  | | |
| No diabetes | 31413 | 506 | 1.6% | Reference | | | | |
| Diabetes | 16422 | 326 | 2.0% | 1.23 | 1.07-1.42 | 0.004 |  |  |
| **Acute renal failure** |  |  |  |  |  |  | | |
| No diabetes | 31413 | 163 | 0.5% | Reference | | | | |
| Diabetes | 16422 | 180 | 1.1% | 2.08 | 1.68-2.59 | <0.001 | | |
| **Acute ischaemic stroke** |  |  |  |  |  |  | | |
| No diabetes | 31413 | 1584 | 5.0% | Reference | | | | |
| Diabetes | 16422 | 1088 | 6.6% | 1.18 | 1.08-1.28 | <0.001 | | |
| **Acute myocardial infarction** |  |  |  |  |  |  | | |
| No diabetes | 31413 | 389 | 1.2% | Reference | | | | |
| Diabetes | 16422 | 269 | 1.6% | 1.29 | 1.1-1.51 | 0.002 | | |
| **Acute pulmonary embolism** |  |  |  |  |  |  | | |
| No diabetes | 31413 | 119 | 0.4% | Reference | | | | |
| Diabetes | 16422 | 59 | 0.4% | 0.97 | 0.71-1.33 | 0.86 | | |

Abbreviations: OR, odds ratio; CI, confidence interval

*Adjusted for sex, age, obesity, modified Charlson comorbidity index, and year of admission

**Supplementary Table 13.** Independent association between diabetes and various post-hip fractures outcomes (excluding patients with a history of cancer)

|  | **Crude HR (95% CI)** | **p** | **Adjusted HR (95% CI)** | **p** |
| --- | --- | --- | --- | --- |
| Prolonged hospitalization | 0.99 (0.96-1.01) | 0.26 | 0.98 (0.96-1.01) | 0.18* |
| Mortality during hospitalization | 0.90 (0.81-1.01) | 0.08 | 0.93 (0.83-1.04) | 0.21* |
| Mortality within 1 year | 1.11 (1.06-1.16) | <0.001 | 1.09 (1.04-1.15) | <0.001* |
| Initiation of anti-osteoporosis treatment in 2 years (n=45756) | 1.03 (0.98-1.07) | 0.22 | 0.99 (0.95-1.04) | 0.66^@^ |
| Recurrent fractures in 2 years | 1.03 (0.96-1.11) | 0.39 | 1.02 (0.95-1.10) | 0.63^#^ |

Abbreviations: HR, hazards ratio; CI, confidence interval

*adjusted for sex, age, obesity, modified Charlson comorbidity index, and year of admission

^@^adjusted for sex, age, obesity, modified Charlson comorbidity index, year of admission, and history of major osteoporotic fractures

^#^ adjusted for sex, age, obesity, modified Charlson comorbidity index, year of admission, history of osteoporosis treatment and history of major osteoporotic fractures
